# Digital Cognitive Behavioral Therapy for Older Adults With Symptoms of Depression: Feasibility Cohort Study

**DOI:** 10.2196/83316

**Published:** 2026-07-03

**Authors:** Khadicha Amarti, Mieke H J Schulte, Annet Kleiboer, Claire Rosalie van Genugten, Mardien Oudega, Artur Rocha, Heleen Riper

**Affiliations:** 1 Department of Clinical, Neuro- and Developmental Psychology, Clinical Psychology Section VU University Amsterdam The Netherlands; 2 Department of Psychology, Education and Child Studies, Centre for Substance and Addiction Research (CESAR), Erasmus University Rotterdam Rotterdam The Netherlands; 3 Department of Psychiatry Amsterdam UMC, location Vrije Universiteit Amsterdam Amsterdam The Netherlands; 4 GGZ inGeest Specialized Mental Health Care Amsterdam The Netherlands; 5 Technology and Science Institute for Systems and Computer Engineering Porto Portugal

**Keywords:** feasibility, depressive disorder, older adults, online intervention, internet-based cognitive behavioral therapy

## Abstract

**Background:**

Depressive symptoms are common among older adults and can significantly impact their quality of life. However, many older adults face barriers to accessing psychological treatment. Internet-based cognitive behavioral therapy (iCBT) is a promising alternative to face-to-face treatments, but its feasibility among older adults has been less extensively studied than in adult populations.

**Objective:**

This study evaluated the feasibility of guided iCBT for adults aged 55 years and older with mild to moderate depressive symptoms recruited from the general population.

**Methods:**

This study is a feasibility study with a single-group, pretest-posttest design (n=21), in which all participants received guided iCBT for 8 weeks. Assessments were conducted at baseline (T0) and after the intervention (T1). The primary outcome was feasibility, conceptualized as satisfaction, usability, engagement, and uptake of iCBT. Secondary outcome measures included depression severity, working alliance, and technical alliance.

**Results:**

Participants were mostly highly educated (13/21, 61.9%), female (18/21, 85.7%), had an average age of 59.85 (SD 4.19; range 55-68) years, and reported moderate digital literacy. Feasibility outcomes indicated high satisfaction and engagement and moderate usability. Working alliance was rated as good by both participants and coaches, and technical alliance was rated as moderate by the participants. There was a nonsignificant modest decrease in depressive symptoms (Cohen *d*=0.47). Of the 20 participants who started the intervention, all completed the first 2 modules, but completion declined across the remaining 6 modules, with only 1 (5%) participant completing all modules.

**Conclusions:**

This study found that guided iCBT has the potential to be a feasible option for older adults experiencing depressive symptoms, with participants reporting generally positive satisfaction, moderate engagement, and a moderate therapeutic bond with their coaches. However, below-average usability ratings and a moderate technical alliance suggest that some aspects of the platform require improvement. Future research should focus on improving usability and adherence, as well as testing the intervention in a larger and more diverse population.

## Introduction

Depressive symptoms, such as sadness, loss of interest, or a sense of emptiness, are common among older adults and can significantly impact their daily functioning and overall quality of life [1]. According to the World Health Organization (2023), approximately 14% of people aged 60 years and older are currently living with a mental disorder, with depression being one of the leading contributors to disability worldwide. Depressive symptoms in older adults often manifest through somatic complaints (eg, fatigue, sleep issues, or physical discomfort), cognitive difficulties, and feelings of loneliness or social disconnection [2,3]. These age-specific symptoms complicate the recognition and diagnosis of depression in older adults. Symptoms may be mistaken for normal aging or overshadowed by comorbid chronic illnesses, such as cardiovascular disease or Alzheimer disease, leading to underdiagnosis and undertreatment [4].

Despite these challenges, older adults benefit from psychotherapy, medication, and lifestyle interventions for the treatment of depression [5]. Among psychological treatments, cognitive behavioral therapy (CBT) has demonstrated effectiveness in reducing both primary depressive symptoms and associated secondary symptoms, such as physical discomfort and reduced quality of life [6,7]. However, many older adults face barriers to accessing face-to-face psychological treatment due to mobility issues, limited availability of specialized care for older adults, and/or high health care costs [7,8].

Digital mental health interventions, particularly internet-based cognitive behavioral therapy (iCBT), have shown promise in overcoming common barriers to traditional treatment, such as stigma, cost, limited service access, and physical mobility constraints [9-12]. iCBT) has been extensively studied and proven effective for reducing symptoms of depression and anxiety in the general adult population [13,14]. Both guided and unguided formats exist. Guided iCBT, where participants receive human support, typically achieves higher adherence and clinical effectiveness in individuals with moderate to severe depression compared with unguided interventions. However, in cases of mild depression, guided iCBT does not consistently outperform unguided interventions [15-17].

Despite the growing interest in iCBT as a treatment for older adults with depression and anxiety, its applicability and effectiveness remain insufficiently studied. Available studies consisted of feasibility or pilot studies, with few randomized controlled trials, often conducted in clinical populations with more severe symptoms. As a result, it is unclear how generalizable these findings are to older adults in the community, who may have milder symptoms and different needs. Existing studies, including Staples et al [18], indicate that older adults can benefit from digital CBT–based interventions, but these studies vary widely in design, population, and outcome measures. Guided iCBT has been shown to reduce depressive symptoms and is often perceived positively by older adults, who value its flexibility and convenience [10,19-24].

However, systematic reviews and meta-analyses emphasized that the evidence base is still in its infancy and largely focused on clinical samples [25-28]. Another underexplored issue is the lack of age-specific adaptations in iCBT, despite indications that tailoring interventions for older adults can improve usability, engagement, and outcomes. For instance, tailored iCBT has been shown to yield significant improvements in depressive symptoms, highlighting the potential of personalized approaches [27,29].

To address these gaps, this study evaluated the feasibility of Moodbuster E-MODEL, a guided online CBT platform, in older adults aged 55 years and older with mild to moderate depressive symptoms recruited from the general population. The primary aim was to assess the feasibility of this guided iCBT format, operationalized through measures of satisfaction, usability, and engagement. Secondary outcomes included changes in depressive symptom severity and the quality of technical and therapeutic alliances.

## Methods

### Design

This study is a feasibility study with a single-group, pretest-posttest design (n=21), in which all participants received a guided iCBT intervention for 8 weeks. Assessments were conducted at baseline (T0) and after the intervention (T1; 8 weeks after baseline). Full details of the study protocol have been published elsewhere [30]. The study protocol included 2 intervention conditions: guided iCBT and blended iCBT (a combination of online sessions and face-to-face support). However, this paper focuses exclusively on the guided iCBT condition.

### Participants

Participants were recruited from the general population through the Link2Trials BV online recruitment platform, which served as the primary setting for study enrollment, between September 2022 and May 2023. Link2Trials BV is a clinical research organization specialized in patient recruitment and adherence services for clinical trials. Participants were included if they (1) were aged 55 years or older, (2) had mild to moderate depression based on a Geriatric Depression Scale-15 (GDS-15) score between 5 and 10, (3) were able to work with a computer, and (4) had access to a computer with internet. The lower age threshold of 55 years was selected to align with demographic classifications used in the Netherlands, where individuals aged 55 and older are considered part of the “younger older adult” population [31]. Additionally, several Dutch mental health services provide care trajectories for adults aged 55 years and older. Care standards note that depression in older adults may present differently due to frailty, highlighting the need for tailored approaches [32]. While this age cutoff is not based on formal clinical guidelines, it allows for a broader and more inclusive representation of older adults experiencing depressive symptoms [9]. Candidates were excluded from the study if they (1) did not have adequate proficiency in the Dutch language or (2) had suicidal thoughts (a score of 1 or higher on item 9 of the Patient Health Questionnaire 9 [PHQ-9]). Excluded participants received an email detailing the reason for their exclusion. On the advice of the Medical Ethical Committee (METC), participants who were excluded because they had moderate to severe depression were advised to contact their general practitioner. Participants identified as being at risk for suicide were advised to contact the national suicide prevention helpline [33].

### Sample Size

The sample size was determined based on recommendations for feasibility studies, which prioritize evaluating study procedures over efficacy testing [34]. Accordingly, the primary outcomes of this study concerned feasibility aspects such as usability, engagement, and acceptability, and not the estimation of effect sizes or statistical power. While sample size guidelines for feasibility studies vary, a range of 24 to 50 participants is typically recommended to ensure adequate representation [34,35]. Given the variability in participants’ age and the diversity of depressive symptom profiles in older adults in the general population, we aimed to recruit 30 participants.

### Procedures

Participants were recruited through the Link2Trials BV online recruitment platform. After expressing interest via the platform, potential participants were contacted by the research team and sent an information letter along with a printed informed consent form by post. Those who were willing to take part in the study were asked to sign and return the consent form by post. The procedure of consent via the post was required by the METC, which mandated a physical signature for ethical approval. Upon receiving the signed informed consent by post, participants were sent a link to the online screening questionnaire via a secure electronic data capture system (Castor). Eligible participants received a link to the online baseline (T0) questionnaire. Once the baseline questionnaire was filled out, participants were assigned a trained coach who provided them with feedback during the intervention. These coaches were master’s-level psychology students who had received training in delivering guided iCBT interventions for older adults and were supervised by a licensed psychologist to ensure adherence to the intervention guidance protocol. The login details for Moodbuster (the online platform) and a manual on the use of the platform were shared with the participants through secure email. The posttreatment assessment (T1) was sent to the participants 8 weeks after the baseline assessment (T0). Participants received a €30 (US $34.84) voucher after completing the postassessment questionnaire, regardless of the time spent on the intervention.

### Intervention

Moodbuster 2.0, developed and tested through several European projects [36-38], is an online and mobile research platform designed for the delivery of online interventions. It includes both a patient and therapist portal and can be used in an unguided, guided, or blended format across various settings. For this study, Moodbuster 2.0 was tailored to better meet the needs of older adults experiencing depressive symptoms. The intervention content was shortened, and the language used throughout the modules was made age appropriate by using examples of everyday situations relevant to the age group, such as physical health changes or coping with retirement. In addition, video content was altered by featuring older adults and professionals discussing depression and CBT principles in a relatable manner.

Moodbuster 2.0 for older adults included 7 web-based CBT modules covering psychoeducation, behavioral activation, cognitive therapy, problem-solving, exercise promotion, sleep hygiene promotion, and relapse prevention. The module on sleep was included as sleep problems are a common contributor to persistent depressive symptoms in this age group [39]. Participants were advised to complete one module per week, starting with psychoeducation, followed by behavioral activation and cognitive therapy, while the other modules were used according to the preference of the participant. An online coach provided feedback once a week for each module through a secure messaging system. If participants were inactive or had not engaged with the platform for some time, the coach would proactively send messages to encourage and motivate them to resume the interventions.

### Measurements

All measures were conducted through web-based self-report questionnaires. Table 1 provides an overview of the assessments at each time-point. In addition to the assessments taken from participants, the Working Alliance Inventory-Short Form (WAI-SF) questionnaire was also filled out by the coaches after the assessment.

**Table 1:** Overview of the outcome measures and instruments assessed in a pilot feasibility study of guided internet-based cognitive behavioral therapy for older adults with depressive symptoms in the Netherlands.

Questionnaire		Aim	Screening	Baseline	Postintervention assessment
**Screening**					
	GDS-15^a^	Depressive symptoms	✓		
	Item 9 of PHQ-9^b^	Suicidal ideation	✓		
**Feasibility**					
	CSQ-I^c^	Satisfaction			✓
	SUS^d^	Usability			✓
	TWEETS^e^	Engagement			✓
**Secondary outcomes**					
	PHQ-8^f^	Depressive symptoms		✓	✓
	WAI-TECH-SF^g^ (therapist version and patient version)	Technical alliance			✓
	WAI-SF^h^ (therapist version and patient version)	Working alliance^i^			✓
**Other measures**					
	DHLI^j^ —operational skills subscale	Digital health literacy—operational skills	✓		
	DHLI—adding content skills subscale	Digital health literacy—adding content	✓		

^a^GDS-15: Geriatric Depression Scale.

^b^PHQ-9: Patient Health Questionnaire-9.

^c^CSQ-I: Client Satisfaction Questionnaire.

^d^SUS: System Usability Scale.

^e^TWEETS: Twente Engagement with eHealth Technologies Scale.

^f^PHQ-8: Patient Health Questionnaire-8.

^g^WAI-TECH-SF: Working Alliance Inventory for Online Interventions-Short Form.

^h^WAI-SF: Working Alliance Inventory-Short Form.

^i^Administered to both participants and coaches.

^j^DHLI: Digital Health Literacy Instrument.

### Screening

#### Depression Severity

Depression severity was assessed using the GDS-15 [40], an instrument specifically designed for older adults. The GDS-15 minimizes the influence of somatic symptoms and uses a simple yes or no response format with low cognitive burden, thereby reducing confounding by physical comorbidity and age-related changes that may affect scores on general adult depression scales such as the PHQ-9 or the Beck Depression Inventory. The GDS-15 consists of 15 items with the option to respond yes or no. Total scores range from 0 to 15, with higher scores indicating a higher severity of depression. The cutoff for mild to moderate depression is a score between 5 and 10. The GDS-15 has been found to be a reliable and valid instrument (Cronbach α=0.83-0.88) [41].

#### Suicidal Ideation

Participants were screened for suicidal ideation using item 9 of the Patient Health Questionnaire-9 (PHQ-9) [42]. This item assesses thoughts of being better off dead or self-harm over the past 2 weeks, with response options ranging from 0 (“not at all”) to 1 (“several days”), 2 (“more than half the days”), and 3 (“nearly every day”). Higher scores indicate higher suicidal ideation. It has a strong predictive validity [43] for suicide attempts and deaths regardless of age.

### Primary Outcome Measures

#### Satisfaction

User satisfaction was measured with the Client Satisfaction Questionnaire adapted to internet-based interventions (CSQ-I) [44]. The CSQ-I consists of eight 4-point Likert scale items with response options ranging from 1 (“does not apply to me”) to 4 (“applies to me”). Total scores range from 8 to 32, with higher scores indicating greater satisfaction. Scores of 20 to 25 indicate “good” satisfaction. The CSQ-I has been found to be a reliable instrument (Cronbach α=0.87) [44].

#### Usability

Usability was assessed using the System Usability Scale (SUS) [45]. The SUS consists of ten 5-point Likert scale items, with response options ranging from 0 (“strongly disagree”) to 4 (“strongly agree”). Total scores are converted to a 0 to 100 scale, where higher scores indicate greater usability. The SUS is a reliable instrument (Cronbach α=0.90), with scores above 68 indicating “good” usability [46].

#### Engagement

Engagement was measured using the Twente Engagement with eHealth Technologies Scale (TWEETS) [47], a 9-item self-reported questionnaire. The TWEETS evaluates engagement in 3 subscales: behavioral, cognitive, and affective engagement. Behavioral engagement assesses the extent to which the technology is integrated into the user's daily routine and the effort required to use it. Cognitive engagement relates to the user's perception of the technology's usefulness in achieving health-related goals and the motivation it provides. Affective engagement captures the user's emotional responses to the technology, such as enjoyment and personal relevance [48]. There are 3 versions of the TWEETS available, namely expected engagement, current engagement, and past engagement. In this study, past engagement was assessed. Responses were recorded on a 5-point Likert scale, with total scores ranging from 0 to 40, where higher scores indicate greater engagement. The TWEETS has demonstrated being a valid tool with good psychometric qualities (Cronbach α=0.87) [48].

#### Uptake

Uptake refers to the degree to which a participant engages with the content of the intervention by using or not using the intervention [49]. Uptake of the intervention was measured through logfile analysis, tracking logins, time spent on the platform, and modules started and completed [50].

### Secondary Outcome Measures

#### Depression Severity

Depression severity was assessed using the Patient Health Questionnaire-8 (PHQ-8) [51]. The PHQ-8 was used instead of the PHQ-9 because the PHQ-8 omits the suicidal ideation item, which was already assessed during screening. The PHQ-8 consists of 8 items scored on a 0 to 3 scale, with total scores ranging from 0 to 24. Higher scores indicate greater depression severity, categorized as mild (5-9), moderate (10-14), moderately severe (15-19), and severe (20-24). The PHQ-8 has been found to be a reliable and valid instrument (Cronbach α=0.82) [51,52].

#### Technical Alliance

Technical alliance was assessed with the Dutch translation of the Working Alliance Inventory for Online Interventions-Short Form (WAI-TECH-SF) [53]. This 12-item questionnaire evaluates alliance with the online platform across 3 dimensions (goals, tasks, and bond), using a 7-point Likert scale. Total scores range from 12 to 84, with higher scores indicating a stronger technical alliance. The WAI-TECH-SF has been found to be a reliable instrument (Cronbach α=0.97) [53].

#### Working Alliance

Working alliance was measured using the WAI-SF [54], which assesses therapeutic alliance across 3 dimensions: goals, tasks, and bond. The patient version consists of 12 items with responses on a 5-point Likert scale, ranging from 1 (“never or rarely”) to 5 (“very often”). The therapist version consists of 10 items with responses on a 5-point Likert scale ranging from 1 (“never”) to 5 (“always”). The WAI-SF has been demonstrated to be a reliable and valid instrument (Cronbach α=0.91) [55].

### Other Measures

Digital literacy is the degree of a person’s knowledge, comfort, and perceived skills with a digital instrument such as a computer [56]. Digital literacy was measured using 2 subscales of the Digital Health Literacy Instrument (DHLI), developed by van der Vaart and Drossaert [56]. The 2 subscales that were used were “operational skills” and “the ability to add content.” The subscale “operational skills” assesses an individual’s ability to use basic digital tools, such as operating a computer or navigating the internet. The subscale “adding content” evaluated a person’s capacity to create and share content, such as posting messages or uploading information [56]. Each subscale consists of 3 items rated on a 4-point Likert scale, with response options ranging from 1 (“very easy”) to 4 (“very hard”), with total scores on both subscales ranging from 3 to 9. Higher scores indicate lower proficiency in operational skills and the ability to add content. The operational skills subscale demonstrated good reliability (Cronbach α=0.77), while the adding content subscale showed excellent reliability (Cronbach α=0.89) [56].

### Analysis

Data analyses were performed using SPSS (version 27; IBM Corp) [57]. Descriptive analyses were used to describe demographic characteristics, digital literacy at baseline, feasibility of the platform for usability, satisfaction, and engagement, as well as working alliance and technical alliance at after the assessment. To assess the difference in depression severity between the preintervention and postintervention measurements, a 2-tailed, paired sample *t* test was performed. The significance level used was *P*<.05. Effect sizes were calculated using Cohen *d*, with thresholds of 0.2, 0.5, and 0.8 interpreted as small, medium, and large effects, respectively [58].

### Ethical Considerations

The study was reviewed and approved by the METC of the VU University Medical Center Amsterdam (2021.0435). All procedures were conducted in accordance with the Declaration of Helsinki. Written informed consent was obtained from all participants before participation. Participant data were processed in a coded and deidentified manner and stored securely in accordance with institutional and General Data Protection Regulation regulations; only authorized members of the research team had access to the data. Participants received a €30 (US $34.84) voucher as compensation for their participation. The reporting adhered to the CONSORT (Consolidated Standards of Reporting Trials) 2010 extension for pilot and feasibility studies to ensure methodological transparency [59].

## Results

### Participants

**Figure 1:**
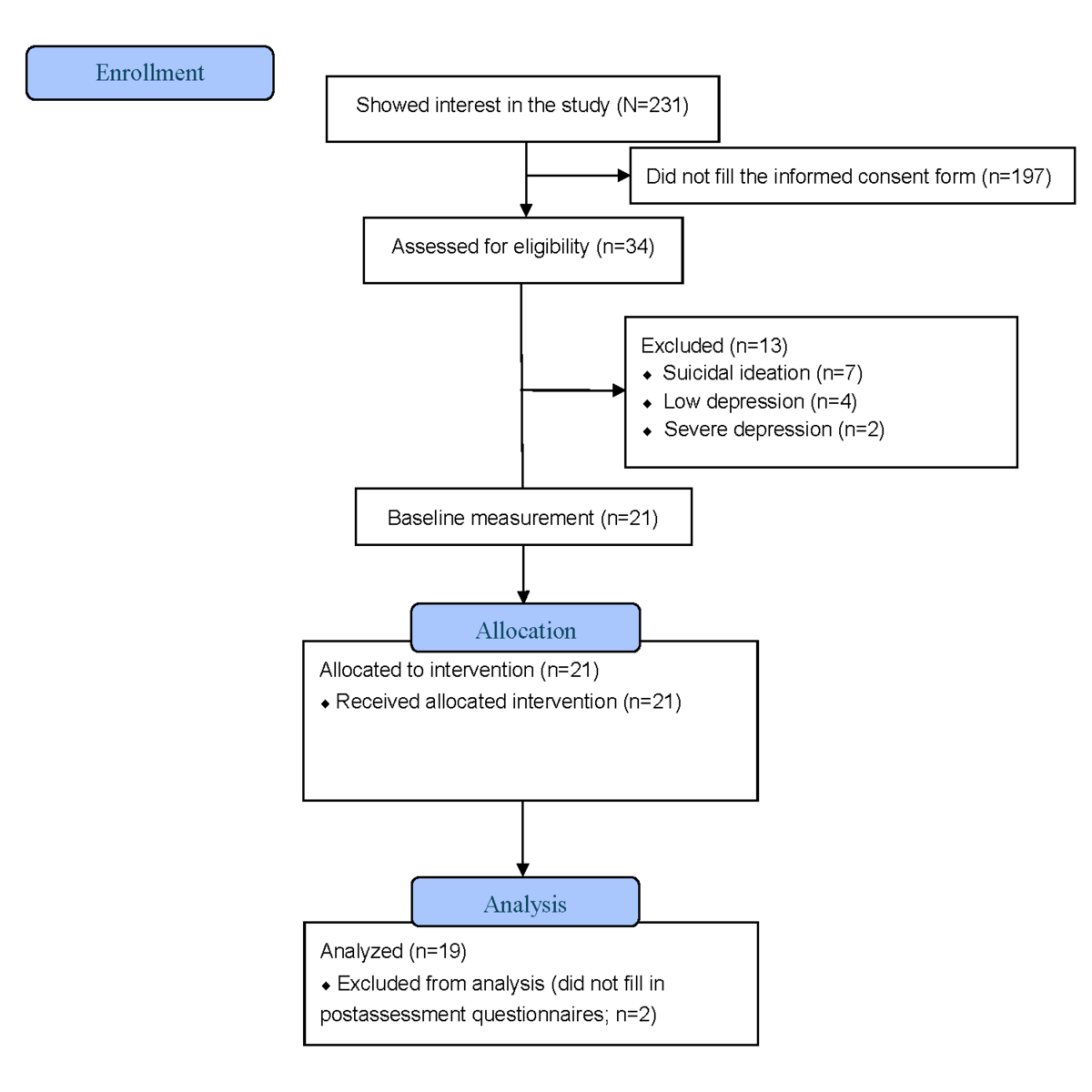
CONSORT (Consolidated Standards of Reporting Trials) flow diagram of participant recruitment, allocation, and retention in a pilot feasibility study of guided internet-based cognitive behavioral therapy for older adults with depressive symptoms.

Of the 231 potential participants (mean age 64.8, SD 6.5 years; n=162, 70.1% female), only 34 (14.7%) signed the paper-based informed consent form and were assessed for eligibility, of which 21 (61.8%) were eligible to take part (mean 7.76, SD 2.14). The CONSORT flowchart diagram of the study is shown in Figure 1. After 8 weeks, 90.5% (19/21) of the included participants completed the postassessment questionnaires.

The baseline characteristics of our participants are summarized in Table 2. The group was predominantly female (18/21, 85.7%), had an average age of 59.85 (SD 4.19; range 55-68) years, was highly educated (13/21, 61.9%), and all but one (20/21, 95.2%) were born in the Netherlands. Their marital status was mixed, and most of them had children (16/21, 76.2%), and all were in contact with them. Regarding their digital literacy, they subjectively reported their operational skills at 3.81 (on a scale from 3 to 9), which we interpret as “very good” as a higher score indicates lower skills (Table 2). However, they scored their ability to add content with an average score of 5.48 (SD 1.91, on a scale from 3 to 9), which is considered “moderate.” Concerning the level of depression, participants showed an average score of 8.61 (SD 4.72) on the PHQ-8, which falls within the range of mild depression.

**Table 2:** Baseline demographic and clinical characteristics of participants enrolled in a pilot feasibility study of guided internet-based cognitive behavioral therapy for older adults with depressive symptoms (n=21).

		Study sample
**Sex, n (%)**		
	Female	18 (85.7)
	Male	3 (14.3)
Age (years), mean (SD)		59.85 (4.19)
**Educational level, n (%)**		
	Primary education	0 (0)
	Secondary education	8 (38.1)
	Higher education	13 (61.9)
	Other	0 (0)
**Marital status**		
	Single	7 (33.3)
	Divorced or widowed	3 (14.3)
	Relationship	2 (9.5)
	Married	9 (42.9)
**Country of birth, n (%)**		
	The Netherlands	20 (95.2)
	Other	1 (4.8)
**Children, n (%)**		
	Yes	16 (76.2)
	No	5 (23.8)
**Contact with children, n (%)**		
	Yes	16 (76.2)
	No	0 (0)
	Not applicable	5 (23.8)
**Daily routine, n (%)**		
	Working	7 (33.3)
	Volunteering	1 (4.8)
	Other	13 (61.9)
**Digital health literacy (DHLI^a^), mean (SD)**		
	Operational skills	3.81 (1.20)
	Adding content	5.48 (1.91)
Baseline depression severity (PHQ-8^b^), mean (SD)		8.61 (4.72)

^a^DHLI: Digital Health Literacy Instrument.

^b^PHQ-8: Patient Health Questionnaire-8.

### Feasibility

Feasibility outcomes are presented in Table 3. Participants reported high satisfaction with the intervention, with a mean score of 20.58 (SD 5.01). Engagement, assessed using the TWEETS, was rated as moderate to high (mean 2.89, SD 0.95) with higher scores in the cognitive engagement subscale (mean 3.11, SD 1.03). However, the average usability score (SUS: mean 54.21, SD 24.00) fell below the accepted threshold (68 or higher).

**Table 3:** Feasibility outcomes, including satisfaction, usability, engagement, and working alliance, reported by participants in a pilot feasibility study of guided internet-based cognitive behavioral therapy for older adults with depressive symptoms (n=19^a^).

		Study sample, mean (SD)
Client satisfaction^b^		20.58 (5.01)
System usability^c^		54.21 (24.00)
**Engagement^d^**		
	Total	2.89 (0.95)
	Behavioral engagement	2.93 (0.88)
	Cognitive engagement	3.11 (1.03)
	Affective engagement	2.65 (1.12)
Technical alliance^e^		45.16 (14.75)
**Working alliance^f^**		3.31 (0.97)
	Therapeutic goals subscale	3.33 (1.22)
	Bond subscale	3.78 (0.99)
	Task subscale	2.83 (1.07)

^a^n=18 for Twente Engagement with eHealth Technologies Scale (TWEETS) and Working Alliance Inventory for Online Interventions-Short Form (WAI-TECH-SF).

^b^Measured with the Client Satisfaction Questionnaire adapted to internet-based interventions.

^c^Measured with the System Usability Scale.

^d^Measured with the TWEETS.

^e^Measured with the WAI-TECH-SF.

^f^Measured with 6 items of the 12 items of the patient Working Alliance Inventory-Short Form; due to unforeseen technical problems, only 2 out of 4 items of each subscale were analyzed.

### Working Alliance Participants and Coaches

The scores of the working alliance (WAI-SF) for participants are shown in Table 3. Participants rated the WAI-SF with their coach as “moderate.” In addition, coaches reported their experience working in alliance with participants. The 4 coaches guided 21 (range 1-10) participants. The coaches scored on average 3.12 out of 5. Subscale scores were highest for the bond dimension (mean 4.46, SD 0.78), followed by the task (mean 2.42, SD 0.60) and therapeutic goals (mean 2.35, SD 0.87).

Participants’ technical alliance (WAI-TECH-SF) is shown in Table 3. Participants rated the technical alliance on average 45.15 (SD 14.75) on a scale from 12 to 84.

### Uptake

Of the 21 participants who completed the baseline, 20 (95.2%) participants started the intervention. On average, participants logged in 19 (SD 19. 49) times, visited 62 (SD 39.89) pages out of the total 133 pages, and spent 117 (SD 159.52) minutes on the platform. Module completion varied considerably across the 8 modules. Nearly all participants completed the introduction (n=20, 95.2%) and psychoeducation (n=19, 90.5%) modules. Engagement then dropped, with 10 (47.6%) participants completing behavioral activation, and 4 (19%) participants completing cognitive therapy. Of the optional modules, 7 (33.3%) participants chose and completed problem-solving, followed by sleep and relapse prevention (n=4, 19%), and only 1 (4.8%) participant completed the exercise module. Only 1 (4.8%) participant finished all modules. Regarding time spent, the problem-solving and cognitive therapy modules showed the most time on average (mean 40.03, SD 26.57 and mean 39.31, SD 26.57 minutes, respectively). These modules also had the highest variation in completion time, showing individual differences in time spent in these modules. Further information about uptake can be found in Table 4.

**Table 4:** Intervention uptake and module completion across the guided internet-based cognitive behavioral therapy intervention for older adults with depressive symptoms (n=21).

Intervention	Total pages per module, n	Module finished, n (%)	Minutes spent per module^a^, mean (SD; range)	Pages visited per module^a^, mean (SD)
Module 1: introduction	8	20 (95.2)	4.54 (2.75; 1-12)	8 (0)
Module 2: psychoeducation	16	19 (90.5)	28.4 (38.55; 2-173)	15.75 (1.12)
Module 3: pleasurable activities	16	10 (47.6)	34.59 (29.42; 2-121)	13.53 (4.49)
Module 4: problem-solving	20	7 (33.3)	40.03 (26.57; 3-93)	18.08 (4.1)
Module 5: cognitive therapy	28	4 (19)	39.31 (26.57; 1-299)	18.25 (10.54)
Module 6: movement	18	1 (4.8)	18.33 (22.9; 1-52)	11.29 (4.57)
Module 7: sleep	15	4 (19)	28.63 (14.84; 13-47)	15 (0)
Module 8: relapse prevention	12	4 (19)	13.4 (3.93; 8-18)	12 (0)

^a^Only includes the participants who have at least started the module.

### Effects of Intervention on Depression Scores

At baseline, the participants reported mild depressive symptoms on the PHQ-8 (mean 8.61, SD 4.72). On average, the depressive symptoms of the group decreased by almost 2 points on the PHQ-8 (mean 6.72, SD 2.93). The paired sample *t* test revealed that this decrease was not significant (t_17_=2.03, 95% CI –0.07 to 3.84; *P*=.06; Cohen *d*=0.47).

## Discussion

The primary aim of this study was to assess the feasibility of a guided online CBT intervention for older adults aged 55 years and older experiencing depressive symptoms. Participants generally reported a good level of satisfaction and moderate to high engagement with the intervention. While most participants began the intervention, adherence varied. Only a small number (1/21, 4.8%) completed all modules, and about one-third (7/21, 33.3%) completed at least 4. Usability of the platform was rated below the standard benchmark, and technical alliance was rated as moderate. Despite this, both participants and coaches indicated a moderate therapeutic alliance, particularly highlighting a good therapeutic bond. A moderate reduction in depressive symptoms was observed, though this change was not statistically significant (*P*=.06).

These findings are consistent with previous research indicating that older adults generally respond positively to guided iCBT, particularly regarding treatment satisfaction and adherence [10,21], as well as reductions in symptoms of anxiety and depression [60,61]. However, usability scores falling below the benchmark of 68 on the SUS [46] suggest that some users encountered challenges navigating or interacting with the platform. Such usability difficulties may have contributed to the low engagement and completion rates observed in the study. Only about half (10/21, 47.6%) of the participants completed 3 or more modules. Older adults may experience higher cognitive load and frustration with digital platforms, for example, due to difficulties with navigation or information processing, which have been shown to negatively affect usability and reduce adherence [62-64]. The strong therapeutic bond reported by both participants and coaches supports previous research highlighting the importance of relational aspects in digital mental health interventions for older adults [53,65]. The moderate technical alliance suggests that participants experienced some technical difficulties forming a sense of connection with the platform itself.

Although depressive symptoms decreased with a medium effect size (Cohen *d*=0.47), the change was not statistically significant, likely due to the small sample size and limited statistical power. Still, a 2-point reduction on the PHQ-8 may have clinical relevance, especially for individuals with subthreshold or moderate symptoms [66]. The completion rate was lower than typically observed in guided iCBT studies, where therapeutic support tends to improve adherence [14,67]. This suggests that barriers such as limited digital literacy, insufficient motivation, or difficulties with the platform’s usability may have influenced engagement [29,68].

One of the study’s strengths lies in its focus on a guided iCBT format, which is particularly relevant for older adults. Guided formats are particularly relevant for this group and have been associated with improved adherence and clinical outcomes [20,21]. By recruiting participants outside clinical settings, this study addresses a gap in the literature, as most iCBT research in older adults has been conducted in clinical samples [27,69]. Additionally, the inclusion of age-relevant content aligns with recommendations to tailor digital interventions to the needs and daily experiences of older adults to enhance acceptability and usability [64].

Despite its strengths, several limitations need to be mentioned. First, although interest in participation was high (N=231), only 34 (14.7%) participants completed the paper-based informed consent and could be assessed for eligibility, resulting in a final sample of 21 (9.1%) participants. The requirement for a physical signature likely created a logistical barrier and may have discouraged participation, highlighting the need for more accessible, secure digital consent procedures in future studies. Second, although the study targeted older adults, the age range of participants (55-68 years) suggests that we primarily reached the younger part of the older population. This means that we did not reach the broader spectrum of older adults, especially those aged 70 years and older, who may face different needs or digital barriers. Moreover, the sample was relatively homogeneous, predominantly female, highly educated, and born in the Netherlands, which limits the generalizability of the findings. Future studies should aim for larger and more diverse samples to increase the applicability of the results to broader populations.

Although this feasibility study identified challenges related to usability and module completion, it also provided critical information to inform the design of a future randomized controlled trial. Usability may be improved by simplifying platform navigation and offering brief support specifically for older adults before using the intervention, while module completion may benefit from more structured or proactive coach contact. In addition, future research could explore whether unguided iCBT may be effective for older adults with mild depressive symptoms, offering a scalable alternative to guided interventions, as suggested by prior research in younger populations [16,17]. Together, these adjustments can inform the design of a future randomized controlled trial evaluating the effectiveness of digital interventions for older adults. In conclusion, findings from this feasibility study indicate that guided iCBT has the potential to be a feasible treatment option for older adults with depressive symptoms. While participants were generally satisfied and engaged, issues related to usability and low module completion indicate the need for improvement. Future research should focus on improving platform usability, adherence, and testing the intervention in larger, more diverse samples. With further development and evaluation, guided digital interventions could become a more accessible and scalable option to address the mental health needs of aging populations.

## Data Availability

The datasets generated and/or analyzed during this study are not publicly available due to privacy and ethical restrictions but are available from the corresponding author on reasonable request.

## Appendix

Multimedia Appendix 1 CONSORT 2010 checklist.

## Abbreviations

CBT: cognitive behavioral therapy
CONSORT: Consolidated Standards of Reporting Trials
CSQ-I: Client Satisfaction Questionnaire adapted to internet-based interventions
DHLI: Digital Health Literacy Instrument
GDS-15: Geriatric Depression Scale-15
iCBT: internet-based cognitive behavioral therapy
METC: Medical Ethical Committee
PHQ-8: Patient Health Questionnaire-8
PHQ-9: Patient Health Questionnaire-9
SUS: System Usability Scale
TWEETS: Twente Engagement with eHealth Technologies Scale
WAI-SF: Working Alliance Inventory-Short Form
WAI-TECH-SF: Working Alliance Inventory for Online Interventions-Short Form
